# A Case Report of Hepatitis E Infection Leading to Acute Liver Failure and Transplantation

**DOI:** 10.4137/ccrep.s845

**Published:** 2008-12-02

**Authors:** P. Paskaran, A.U. Murugananthan, V. Manglam, J. Arnold, A. Pal

**Affiliations:** 1 Department of Gastroenterology, Ealing Hospital NHS Trust, London.; 2 Imperial College School of Medicine, London.

Historically, Hepatitis E infection has been thought to follow a benign clinical course. We report a case of Acute Hepatitis E infection in a non-pregnant female, resulting in the development of Acute Liver Failure necessitating a liver transplant.

Our patient was a 31 year-old Somalian born lady. She had lived in England for more than 15 years but had lived for a period of 8 months in Canada before returning to England two months prior her presentation. She lived in West London which is an ethnically diverse population. This patient initially presented to the Accident and Emergency Department with jaundice but was discharged to be followed-up in clinic. Outpatient blood investigations were done on two further occasions before the patient was finally admitted with steadily rising bilirubin levels. There was no history of travel to countries other than Canada and the U.K., no history of illicit drug use, herbal remedies, or blood transfusions. She had been married for 9 years with three children. There was also no history of any recent travel to any Hepatitis E endemic regions by any of her family members.

On examination at presentation, she was markedly icteric with non-tender hepatomegaly. She was haemodynamically stable with no clinical evidence of hepatic encephalopathy. A full liver screen was requested and included tests for Hepatitis A, B and C, EBV, CMV, Autoantibodies, Ferritin, Caeruloplasmin, Immunoglobulins, α1-antitrypsin as well as screening for Acetaminophen (Paracetamol) poisoning. An ultrasound of the liver was performed and showed patent, non-occluded liver vasculature. After 6 days of stable observation on a general medical ward she deteriorated abruptly and became encephalopathic. Her blood results are summarized in Table 1.

She was intubated, ventilated and transferred to the Regional Liver Intensive Care Unit where she was listed super-urgently for orthotopic liver transplantation. She was operated on Day 8 post-admission as she met the transplant criteria of jaundice (Bil >300), coagulopathy (PT >50s) and jaundice to encephalopathy time (J-E time >7 days). Immediate graft function post-transplant was very good and she had an unremarkable recovery.

Explant liver biopsy showed regions of extensive centrilobular and mid-zonal hepatocyte loss with acute haemorrhage, ductular reaction, cholestasis, and slight centrilobular stromal collapse, interspersed with regions of hepatocellular hyperplasia. A mixed but predominantly lymphocytic infiltrate was seen throughout the lobule.

The eventual diagnosis of acute hepatitis E infection was only confirmed *post* transplantation with Enzyme-Linked ImmunoSorbent Assay (ELISA) performed on the patient’s blood taken at the time of admission and kept in refrigeration. Tests for viral load, such as Reverse Transcriptase Polymerase Chain Reaction (RT-PCR) were not performed.

## Discussion

Hepatitis E virus (HEV), previously known as non-A, non-B hepatitis is a self-limiting, waterborne or enterically-transmitted acute viral hepatitis.^1^ Recent reported infections among surgical trainees in the U.K. also suggest possible zoonotic modes of transmission.^2^ HEV is an icosahedral, non-enveloped single stranded RNA virus that is structurally similar to viruses of the Hepeviradae family.^3^ The incubation period following exposure to HEV ranges from 3 to 8 weeks, with a mean of 40 days. Symptomatic HEV infection is most common in young adults aged 15–40 years which is mostly self-limiting. However, youth and female sex are seen as poor prognostic factors as is pregnancy where acute liver failure can result in the third trimester.^4^,^5^ Progression to chronic infection has not been reported.

Infection with this virus was first documented in 1955 during an outbreak in New Delhi, India.^6^ The highest incidence of HEV infection is in Asia, Africa, Middle East, and Central America band HEV is the second most common cause of sporadic hepatitis in North Africa and the Middle East.^7^ In the largest reported outbreak, over 100,000 individuals were afflicted in the Xinjiang region of China between 1986 and 1988.^8^ However, Hepatitis E infection is also found in regions outside the equatorial belt and is probably more widespread in industrialized countries than generally believed.^9^ A Japanese review of patients diagnosed with new-onset autoimmune hepatitis retrospectively showed that a significant proportion actually had Hepatitis E infection when cryogenically preserved serum samples were analyzed years later.^10^

Despite the increasing prevalence of Hepatitis E in non-endemic regions due to globalization and travel, the vast majority of cases still present within the equatorial belt. Of 186 cases of Hepatitis E infection in the U.K. between 1996 and 2003, only 17 of these cases were *not* associated with travel abroad to the traditional endemic areas of SouthEast Asia, Northern Africa, Central America and the Middle East.^11^ Furthermore, all patients in this population were over the age of 55 and were predominantly male (76% vs 24%). Only two patients progressed to acute liver failure.

Our diagnosis of hepatitis E was confirmed post Orthotopic Liver Transplant with serum ELISA IgM HEV on stored samples. The diagnosis of HEV can also be based upon the detection of the HEV genome in serum or faeces by PCR. IgM rheumatoid factor in the serum can cause a false positive serum HEV IgM and therefore HEV IgA, if available, increases the specificity of the diagnosis.^12^

This patient is unusual in that she is a young female who developed fulminant hepatic failure from Hepatitis E infection which required an Orthotopic Liver transplant. Although pregnancy has been shown to be associated with a higher incidence of progression to Acute Liver Failure, this did not apply here.^4^,^13^ Additionally, her ANA titres rose during the period out of hospital when her bilirubin was continuing to rise. Importantly, her Smooth Muscle, Rheumatoid Factor, Anti-Mitochondrial and LKM antibodies were negative. Autoantibodies have been shown to be present in Acute Liver Failure, but some have postulated that their presence may actually indicate the presence of an underlying autoimmune mechanism contributing to the development of Acute Liver Failure.^14^ However, in the case of our patient she did not have sufficient features to suggest Autoimmune hepatitis as defined by Revised Autoimmune Hepatitis Scoring System put forward by the International Autoimmune Hepatitis Group in 1999.^15^

Although the link to autoimmunity may be tentative, it may well be that other factors, yet to be determined, are involved in the progression to Acute Liver Failure in the context of Hepatitis E infection given that it is statistically a relatively rare occurrence. This case highlights the fact that jaundiced patients diagnosed with Hepatitis E should be followed up by physicians or general practitioners until resolution occurs.

## Figures and Tables

**Table 1:** Summary of Patient’s Liver function tests during course of Illness.

	Day 1	Day 7	Day 20	Day 22	Day 28
Bilirubin (3–21 μmol/l)	122	156	360	399	433
ALP (40–150 IU/l)	133	154	140	135	128
ALT (0–55 IU/l)	500	494	996	1027	535
GGT (9–36 IU/l)	83	81	32	28	21
Albumin (35–50 g/L)	33	32	24	25	23
PT (11.5–16 s)	14.9	14.1	–	35.5	61
ANA		1:160			

Her ANA titer in 1999 was 1:40 (speckled pattern).

Her SMA, RF, AMA and LKM antibodies were negative.
